# Neutralizing Anti-Rituximab Antibodies and Relapse in Membranous Nephropathy Treated With Rituximab

**DOI:** 10.3389/fimmu.2019.03069

**Published:** 2020-01-13

**Authors:** Sonia Boyer-Suavet, Marine Andreani, Maël Lateb, Benjamin Savenkoff, Vesna Brglez, Sylvia Benzaken, Ghislaine Bernard, Patrick H. Nachman, Vincent Esnault, Barbara Seitz-Polski

**Affiliations:** ^1^Service de Néphrologie-Dialyse-Transplantation, CHU de Nice, Université Côte d'Azur, Nice, France; ^2^CRMR SNI, CHU de Nice, Université Côte d'Azur, Nice, France; ^3^Service de Néphrologie, Dialyse et Aphérèse Thérapeutique, CHR Metz-Thionville, Thionville, France; ^4^Laboratoire d'Immunologie, CHU de Nice, Université Côte d'Azur, Nice, France; ^5^Division of Renal Diseases and Hypertension, University of Minnesota, Minneapolis, MN, United States

**Keywords:** membranous nephropathy, rituximab, anti-CD20 monoclonal antibody, immunogenicity, immuno-monitoring

## Abstract

Membranous Nephropathy (MN) is an autoimmune disease associated with antibodies against podocyte proteins: M-type phospholipase A2 receptor (PLA2R1) or thrombospondin type-1 domain-containing 7A (THSD7A) in 70 and 3% of patients, respectively. Antibody titer is correlated with disease activity: rising during active disease and decreasing before remission. Therefore, decreasing PLA2R1-Antibodies titer has become an important goal of therapy. Rituximab a chimeric monoclonal antibody induces remission in 60–80% of primary MN patients. All monoclonal antibodies such as rituximab can elicit antidrug antibodies, which may interfere with therapeutic response. We aim to analyze the relevance of anti-rituximab antibodies on the outcome of MN after a first course of rituximab. Forty-four MN patients were included and treated with two 1 g infusions of rituximab at 2-weeks interval. Anti-rituximab antibodies, CD19 count, and clinical response were analyzed. Then, we (i) analyzed the association of anti-rituximab antibodies at month-6 with response to treatment: remission, relapse and the need for another rituximab course; (ii) confirmed if anti-rituximab antibodies could neutralize rituximab B-cells depletion; and (iii) tested whether anti-rituximab antibodies could cross-inhibit new humanized anti-CD20 therapies. Anti-rituximab antibodies were detected in 10 patients (23%). Seventeen patients received a second rituximab course after a median time of 12 months (7–12), following nine cases of resistance and eight relapses. Anti-rituximab antibodies were significantly associated with faster B-cell reconstitution at month-6 (75 [57–89] vs. 2 [0–41] cells/μl, *p* = 0.006), higher proteinuria 12 months after rituximab infusion (1.7 [0.7; 5.8] vs. 0.6 [0.2; 3.4], *p* = 0.03) and before treatment modification (3.5 [1.6; 7.1] vs. 1.7 [0.2; 1.7] *p* = 0.0004). Remission rate 6 months after rituximab was not different according to anti-rituximab status (*p* > 0.99) but the rate of relapse was significantly higher for patients with anti-rituximab antibodies (*p* < 0.001). These patients required more frequently a second course of rituximab infusions (7/10 vs. 10/34, *p* = 0.03). Anti-rituximab antibodies neutralized rituximab activity in 8/10 patients and cross-reacted with other humanized monoclonal antibodies in only two patients. Three patients with anti-rituximab antibodies were successfully treated with ofatumumab. Anti-rituximab antibodies could neutralize rituximab B cells cytotoxicity and impact clinical outcome of MN patients. Humanized anti-CD20 seems to be a satisfying therapeutic alternative for patients with anti-rituximab antibodies and resistant or relapsing MN.

## Introduction

Membranous Nephropathy (MN) is a renal autoimmune disease defined by sub-epithelial immune complex deposits inducing a dysfunction of the glomerular basement membrane. Most cases are associated with antibodies against podocyte proteins: M-type phospholipase A2 receptor (PLA2R1) or thrombospondin type-1 domain-containing 7A (THSD7A) in 70% and 3% of patients, respectively (1, 2). The pathogenic role of anti-PLA2R1 antibodies (PLA2R1-Ab) is not yet demonstrated, but antibodies titers correlate with disease activity i.e., rising during active phases and decreasing before clinical remission (3). One third of patients enter inyo spontaneous remission while another third progresses to end stage kidney disease (4). High titers of PLA2R1-Ab at diagnosis are associated with poor clinical outcome (5). Reducing anti-PLA2R1 antibody levels is a major goal of treatment.

Rituximab is a chimeric monoclonal antibody including human IgG1 constant regions and a murine anti-human CD20 variable region which can lyse lymphocytes B (CD20+ cells) (6). Rituximab was first used in non-Hodgkin lymphoma treatment (7), but is now used in many auto-immune diseases (8–13) including MN (3, 14–17), with an excellent efficacy and tolerability in comparison to more conventional treatment regimens (18, 19). In fact, rituximab induced clinical remission in 60–80% of patients with primary MN in several non-randomized studies (3, 14–16) and its efficacy was established in a recent controlled study after an extended follow-up (17). Rituximab efficacy increases with regimens using high doses (1 g D0 and D15 i.e., 2 g) with 67% of remission at month-6 vs. low doses (375 mg/m^2^ D0 and D7 i.e., 1.4 g) with 33% of remission at month-6 (20).

While rituximab efficacy seems to be well-established in MN, many factors could modify rituximab response. Rituximab pharmacokinetic is largely variable among patients, related to genetic factors or disease, which could impact on B-cell lysis and clinical response (21–23). However, rituximab may be lost in the urine of nephrotic patients and a close monitoring of rituximab residual level could help to retreat patients underexposed to rituximab after a first line (23, 24). Some cases of resistance after rituximab have been described in lymphoma by a decreasing of CD20 expression after repeated rituximab therapies (25). Moreover, monoclonal antibodies such as rituximab can elicit antidrug antibodies, which may interfere with therapeutic response.

First generation fully murine monoclonal antibodies led to very high levels of antidrug antibodies and the murine constant region of these antibodies interacted poorly with human FcRn on endothelial cells with little recycling and rapid clearance from the body. In second-generation chimeric monoclonal antibodies, such as rituximab, human constant regions replaced the corresponding murine regions, resulting in decreased immunogenicity and increased serum residual levels. Humanized or fully human monoclonal antibodies were later developed to further decrease immunogenicity. For example, IgG antibodies to infliximab developed in about 60% of patients with Crohn's disease blunting treatment response (26). In six multinational trials evaluating bococizumab, a monoclonal antibody targeting PCSK9, antidrug antibodies significantly attenuated the decreasing of LDL cholesterol levels (27). A recent study demonstrated that immunization to rituximab is more frequent in systemic autoimmune diseases (31.1%) than rheumatoid arthritis (8.6%) (28). In pemphigus vulgaris and ANCA-associated vasculitis, patients with anti-rituximab antibodies presented disease relapses (11, 12). In systemic lupus erythematosus (SLE), antibodies to rituximab correlated with poor B-cell depletion and negative outcomes (22), impaired normalization of dsDNA titers and predict infusion-related reactions (29). In previous MN studies, Fervenza et al. detected anti-chimeric rituximab antibodies in 6 of 15 patients but these antibodies were not associated with remissions (16).

The improved patient-outcomes and cost-effectiveness have led to the development of other anti-B cells agents (30). New monoclonal antibodies targeting CD20 are currently studied in non-Hodgkin lymphomas and autoimmune diseases (7, 31), including two humanized IgG1: obinutuzumab and ocrelizumab (Roche®); and a fully-human IgG1: ofatumumab (GSK®).

We aim to monitor development of anti-rituximab antibodies in a cohort of patients treated for primary MN and to assess whether resistance or relapse of MN after rituximab treatment could be associated with the development of anti-rituximab antibodies. We then tested whether new humanized and fully human anti-CD20 monoclonal antibodies could be used as therapeutic alternatives.

## Materials and Methods

### Drug Minimal Cytotoxic Concentration Assessment

We assessed *in vitro* minimal anti-CD20 monoclonal antibody cytotoxic concentration. Anti-CD20 monoclonal antibodies (rituximab, obinutuzumab, ocrelizumab and ofatumumab) at 6.25, 12.5, 25, and 50 ng/ml were incubated with 1.5 × 10^3^ purified B-cells (MACSprep^TM^ HLA B Cell Isolation Kit, Milteny Biotec) for 30 min at room temperature in 60-well Terasaki plates (Dutcher^©^ Strasbourg, France) in duplicates of 1 μl per well. Then, 5 μl per well of standard rabbit complement (Cerdarlane^©^ Ontario, Canada) were added for 45 min at room temperature. Dead cells were then revealed after adding 2.5 μl per well of Fluoroquench AO/EB staining/quench (Ingen^©^ Chilly-Mazarine, France) for 10 min in darkness.

Two blinded independent evaluators read the percentage of dead cells using a fluorescent microscope (Axiovert 100 Carl Zeiss^©^ Göttigen, Germany).

### Patient Population

Patients were included after signing informed consent (NCT02199145). They were recruited in Nice in the Department of Nephrology-Dialysis-Transplantation at Pasteur University Hospital between July 2014 to January 2018. Inclusion criteria were: (a) biopsy-proven MN; (b), idiopathic MN defined by the absence of anti-nuclear antibodies, negative hepatitis B and C serologies, and negative cancer investigations (whole-body CT-scan, gastro-intestinal endoscopy, PSA for men and mammography for women); (c) persistent nephrotic proteinuria (i.e., urinary protein/creatinine ratio >3.5 g/g) after 6 months of maximal antiproteinuric treatment or early deterioration of kidney function, or complications of the nephrotic syndrome; (d) follow-up of at least 1 year.

Patients received two 1 g infusions of rituximab at 2-weeks interval after 6 months of symptomatic treatment and persistent nephrotic syndrome or earlier in cases of kidney failure or thrombosis. Patients did not receive concomitant immunosuppressive treatments except 100 mg of methylprednisolone dose at each rituximab infusion according to protocol. Serum and urine samples were prospectively collected before the first infusion, at months 3, 6, and 12 to measure rituximab serum levels, and anti-PLA2R1 antibodies, CD19 positives cells, serum creatinine and proteinuria. Anti-rituximab antibodies were measured at month-3 and also month-6 due to their delayed ability to be detected. In fact, the assay only measures free anti-rituximab antibodies and before month-6, rituximab is still detected, and circulating antibodies link to the drug.

Remissions were defined according to the 2012 KDIGO guidelines. Complete remission was defined by a proteinuria <0.3 g/g with normal serum albumin levels and preserved kidney function. Partial remission was defined by proteinuria <3.5 g/g with over 50% reduction of proteinuria, increasing, or normalization of albuminemia levels and preserved kidney function (serum creatinine levels increase from baseline below 30%). Remissions were counted at month 6 and subsequently before any treatment modification. A relapse was defined by an increase of proteinuria over 3.5 g/g after remission and an increase of anti-PLA2R1 antibodies for PLA2R1 related MN.

A second course of rituximab was needed for resistance to a first course of rituximab (i.e., persistent anti-PLA2R1 activity for anti-PLA2R1 related MN and active disease, after 1 year of follow-up) or relapse [increasing proteinuria (active disease) after complete or partial remission and positive anti-PLA2R1 activity, for anti-PLA2R1 related-MN].

### Detection of Anti-PLA2R1 and Anti-THSD7A Antibodies

Total IgG anti-PLA2R1 level was measured by ELISA (EUROIMMUN, Germany) and was considered positive above 14 RU. Total IgG anti-THSD7A was detected by indirect immunofluorescence (EUROIMMUN, Germany) at 1:10.

### Measurement of Rituximab by ELISA

Serum rituximab level was measured by ELISA, according to the manufacturer's instructions (LISA- TRACKER, Theradiag^©^ Croissy Beaubourg, France). This assay measures only free rituximab. The limit of detection defined by the manufacturer was 2 μg/ml, with an intrarun variability of 8% and interrun variability of 10%.

### Anti-Rituximab Antibodies Detection

Anti-rituximab antibodies were detected by ELISA according to the manufacturer's instructions (LISA- TRACKER, Theradiag^©^ Croissy Beaubourg, France). This assay measures only free anti-rituximab antibodies. The limit of detection for anti-rituximab antibodies defined by the manufacturer was 5 ng/ml with an intra-run variability being at 9.1% and inter-run variability at 10.6%.

### Neutralization of Anti-CD20 Monoclonal Antibodies by Anti-Rituximab Antibodies *in vitro*

Serum samples incubated in the presence of rituximab were used to test the potential neutralizing effect of anti-rituximab antibodies.

#### *In vitro* Complement-Dependent Cytotoxicity Assay

B-cell cytotoxicity was measured in different conditions as described by Terasaki. Ten microliters of serum from patients with anti-rituximab antibodies were incubated with 10 μl of different anti-CD20 monoclonal antibodies (rituximab, obinutuzumab, ocrelizumab, or ofatumumab) at 50 ng/ml. Sera from healthy donors were used as negative controls. Each sample was pre-incubated for 2 h at room temperature, before adding 1.5 × 10^3^ purified B-cells for a 30-min incubation at room temperature in 60-well Terasaki plates in duplicates of 1 μl per well. Then, 5 μl per well of standard rabbit complement were added for 45 min at room temperature. Dead cells were then revealed after adding 2.5 μl per well of Fluoroquench AO/EB staining/quench for 10 min in darkness. Two blinded independent evaluators estimated the percentage of dead cells using a fluorescent microscope.

#### Antibody-Dependent Complement-Independent Cell Cytotoxicity Assay

After a Ficoll separation, 1.8 × 10^6^ peripheral blood mononuclear cells (PBMC) from healthy donor were incubated overnight at 4°C with 20 μl of anti-CD20 monoclonal antibodies (rituximab, obinutuzumab, ocrelizumab, or ofatumumab) at 50 ng/ml; pre-incubated with 20 μl of serum from patients with anti-rituximab antibodies or from healthy donor diluted at 1:2. All samples were heated at 54°C for 30 min to inhibit complement activity. Cell viability was assessed using 10 μl of tryptan blue added to 90 μl of PBMC incubated in each condition. Numbers of dead and alive cells were counted in four different fields. The cells were washed three times in 3 ml of PBS (Cell Wash BD Biosciences^©^ Erembodegem, Belgium) at 4°C and incubated 30 min in darkness with a panel of antibodies specific for T, B, and NK cells: anti-CD3, anti-CD4, anti-CD8, anti-CD45, anti-CD19, anti-CD16 et CD56 (6-color TBNK Reagent BD Biosciences). Then, lysing Solution was added, and samples were incubated 10 min in darkness. The percentages of the T-lymphocytes (CD3+), B-lymphocytes (CD19+), and NK-cells (CD3- CD19-) were determined using Cytometer BD FACS Canto II.

### Endpoints

We first compared the association of anti-rituximab antibodies at month-6 with response to treatment: remission, relapse and the need for a second course of rituximab. We then confirmed if anti-rituximab antibodies could neutralize rituximab B-cells depletion and tested whether anti-rituximab antibodies could cross-inhibit new humanized anti-CD20 monoclonal antibodies.

### Statistical Analyses

For descriptive statistics, data are presented as median (ranges) (for variables with non-Gaussian distribution) or mean ± standard deviation (for variables with Gaussian distribution). We used the Shapiro-Wilk test to test if a variable has a normal distribution. Comparison of qualitative criteria was performed using Chi-square test or Fisher's exact test (according to the terms of use). Comparison of quantitative variables was performed using the Student *t*-test or Wilcoxon-Mann-Whitney test (according to normal distribution). A *p* < 0.05 indicated statistical significance. Survival curves were calculated using Kaplan-Meier estimates for survival distribution. Statistical analyses were performed using GraphPad Prism 7.0 (GraphPad Software, Inc., San Diego, CA).

## Results

### Population Characteristics

**Figure 1:**
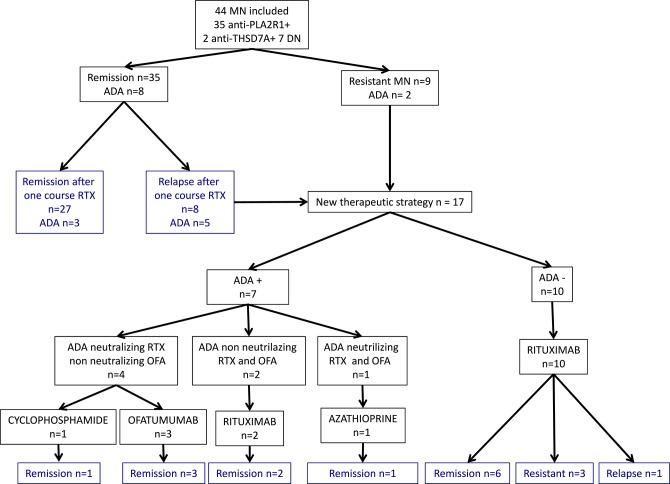
Primary membranous nephropathy cohort and outcomes. Flow chart of 44 primary membranous nephropathy patients and outcome after rituximab therapy. MN, membranous nephropathy; DN, double negative; ADA, Anti-Drug Antibodies; RTX, rituximab; OFA, ofatumumab.

A total of 44 patients with idiopathic MN treated with two perfusions of 1 g-rituximab at 2-weeks interval were included and followed for a median time of 30 months [24–60]: 35 (80%) had anti-PLA2R1 antibodies, two (4%) had anti-THSD7A antibodies, and seven patients (16%) were double negative (Figure 1). Table 1 shows the characteristics of the study cohort. All patients were on Renin-Angiotensin System inhibitor. Remission (partial or complete) was obtained in 35 of 44 patients (79%) in a median time of 3 months [3; 9] and nine patients were resistant to a first rituximab course. Residual serum rituximab level at month 3 was inversely correlated with proteinuria at month 6 (*r* = −0.70; *p* < 0.0001) (Supplementary Figure 1). After remission, eight patients relapsed in a median time of 15 months [10.5; 24]. At least, 17 patients were resistant or relapsed and benefited from a new therapeutic strategy in a median time of 12 months (7–12) (Table 2). Patients who required a second rituximab course exhibited an increase in proteinuria and anti-PLA2R1 antibodies titer before retreatment (Supplementary Figures 2A,B): we observed similar outcomes for relapsing and resistant patients (Supplementary Figures 3, 4). Twenty-seven patients were still in remission after one course of rituximab at last observation.

**Table 1:** Cohort characteristics and outcome data.

**Characteristics**	**Value**
Age (years)	67 ± 15
Gender (female/male)	14/30
**BASELINE CHARACTERISTICS**	
Proteinuria (g/g)	5.9 [4.7–7.8]
Serum creatinine (μmol/l)	107 [85–147]
Serum albumin (g/dl)	2.0 ± 0.7
Anti-PLA2R1 titer (RU/ml)	152 [60–271]
CD19 at M0 (cell/μl)	200 [114–299]
**CHARACTERISTICS AT MONTH 3**	
Proteinuria (g/g)	2.9 [1.2–5.9]
Serum creatinine (μmol/l)	103 [87–143]
Anti-PLA2R1 titer (RU/ml)	3 [0–18]
CD19 (cell/μl)	0 [0–2]
Patients with anti-rituximab antibodies	0 (0%)
Serum rituximab level (μg/ml)	2.27 [0.19–7.5]
**CHARACTERISTICS AT MONTH 6**	
Proteinuria (g/g)	1.7 [0.9–4.5]
Serum creatinine (μmol/l)	104 [84–136]
Anti-PLA2R1 titer (RU/ml)	0 [0–1]
CD19 (cell/μl)	9 [2–70]
Patients with anti-rituximab antibodies	10 (23%)
Serum rituximab level (μg/ml)	0 [0–0]
**CHARACTERISTICS AT MONTH 12**	
Proteinuria (g/g)	0.9 [0.3–2.9]
Serum creatinine (μmol/l)	101 [87–130]
Serum albumin (g/dl)	3.6 ± 0.6
Anti-PLA2R1 titer (RU/ml)	1 [0–7]

**Table 2:** Treatment history for rituximab resistant or relapsing patients.

	**ADA status**	**First line**	**Outcome**	**Second line**	**Outcome**	**Third line**	**Outcome**	**Fourth line**	**Outcome**
Patient 1	+	RTX	Relapse (12)	RTX	Relapse (12)	RTX	Relapse (12)	RTX AZA	PBC Remission (6)
Patient 3	+	RTX	Relapse (6)	RTX	Remission (3)				
Patient 4	+	RTX	Resistant	RTX	Resistant	OFA	Remission (3)		
Patient 5	+	RTX	Resistant	RTX	Resistant	OFA	Remission (3)		
Patient 7	+	RTX	Relapse (48)	OFA	Remission (30)				
Patient 9	+	RTX	Resistant	RTX	Remission				
Patient 10	+	RTX	Resistant	CYC	Resistant				
Patient 11	–	RTX	Resistant	RTX	Remission (6)				
Patient 12	–	RTX	Resistant	RTX	Remission (6)				
Patient 13	–	RTX	Resistant	RTX	Remission (6)				
Patient 14	–	RTX	Resistant	RTX	Remission (6)				
Patient 15	–	RTX	Resistant	RTX	ESKD				
Patient 16	–	RTX	Resistant	RTX	ESKD				
Patient 17	–	RTX	Relapse	RTX	Resistant	RTX	ESKD		
Patient 18	–	RTX	Relapse	RTX	Relapse	RTX	Relapse	RTX	Remission (6)
Patient 19	–	RTX	Relapse	RTX	Relapse	RTX	Relapse	RTX	Remission (6)
Patient 20	–	RTX	Relapse	RTX	Remission				

*ADA, Anti-Drug Antibodies; RTX, Rituximab; AZA, Azathioprine; OFA, Ofatumumab; CYC, Cyclophosphamide. PBC, Primary Biliary Cholangitis*.

### Detection and Assessment of Anti-Rituximab Antibodies

**Figure 2:**
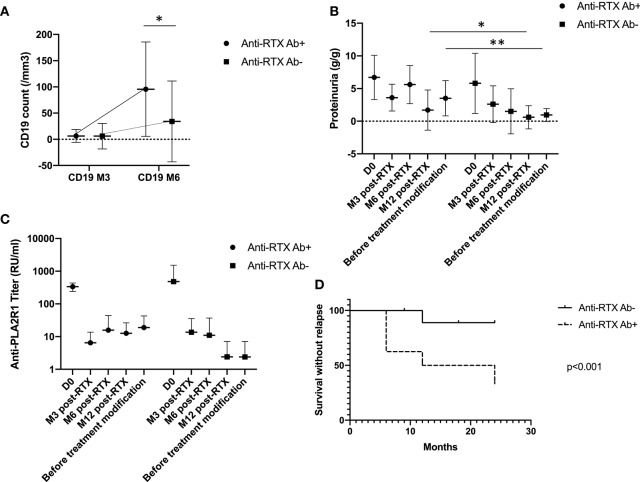
Anti-rituximab antibodies and outcomes. **(A)** CD19+ B-cells evolution according to anti-rituximab antibodies status: initial CD19+ B-cells depletion was seen in all patients. But for patients who developed anti-rituximab antibodies, B-cells recovered earlier. *p = 0.006. **(B)** Proteinuria evolution according to anti-rituximab antibodies status: proteinuria stopped decreasing or increased in patients who developed anti-rituximab antibodies *p = 0.03, **p = 0.004. **(C)** Anti-PLA2R1 antibodies titer evolution according to anti-rituximab antibodies status: anti-PLA2R1 antibodies levels did not decrease or increased following rituximab in patients who developed anti-rituximab antibodies. Before treatment modification anti-PLA2R1 titer tended to be higher in patients with anti-rituximab antibodies (*p* = 0.09). **(D)** Renal survival without relapse within 2 years after rituximab therapy according to anti-rituximab antibodies status: patients with anti-rituximab antibodies exhibited more relapses within 2 years after rituximab therapy (*p* < 0.001).

Anti-rituximab antibodies could be detected at month-6 in 10 patients (23%) (undetectable at month-3) and persisted in nine patients (20%). These patients had similar characteristics at diagnosis but during follow-up patients with anti-rituximab antibodies had higher level of CD19 counts at month 6, since patients with anti-rituximab antibodies showed faster B-cells reconstitution (75 [57–89] vs. 2 [0–41], *p* = 0.006) (Figure 2A), higher proteinuria at month-12 (1.7 [0.7–5.8] vs. 0.6 [0.2–3.4] *p* = 0.03), and before treatment modification (3.5 [1.6; 7.1] vs. 1.7 [0.2; 1.7] *p* = 0.0004) (Figure 2B and Table 3). Remission rate was not different according to anti-rituximab status [8/10 (80%) vs. 27/34 (79%) *p* > 0.99] but relapses were associated with anti-rituximab antibodies [5/10 (50%) vs. 3/34 (9%) *p* = 0.009]. Patients who developed anti-rituximab antibodies required a higher number of rituximab infusions [7/10 (70%) required a second course of rituximab vs. 10/34 (29%) *p* = 0.03] (Table 3). Figure 2 shows evolution of proteinuria, anti-PLA2R1 antibodies titer and CD19+ B-cells rate according to the anti-rituximab antibodies status. Anti-PLA2R1 antibodies levels stop decreasing or increased after month-3 post-rituximab in anti-rituximab antibodies immunized patients and tended to be different before treatment modification (8 [0; 34] vs. 0.5 [0; 15] *p* = 0.09) (Table 3 and Figure 2C).

**Table 3:** Clinical characteristics and outcome in rituximab-immunized or non-immunized patients.

	**Anti-RTX antibodies +** ***n* = 10**	**Anti-RTX antibodies–** ***n* = 34**	***p-*value**
Age (years)	60 ± 13	60 ± 14	0.99
Gender (female/male)	3/7	11/23	0.99
**BASELINE CHARACTERISTICS**			
Proteinuria (g/g)	6.7 [4.7–10.6]	5.8 [4.6–7.7]	0.42
Serum creatinine (μmol/l)	127 [105–151]	107 [78–149]	0.30
Serum albumin (g/dl)	1.9 ± 0.6	2.1 ± 0.7	0.74
Anti-PLA2R1 titer (RU/ml)	148 [72–243]	159 [45–288]	0.99
CD19 (cell/μl)	236 [190–287]	143 [62–290]	0.08
**CHARACTERISTICS AT MONTH 3**			
Proteinuria (g/g)	3.6 [2.3–5.9]	2.6 [0.9–5.9]	0.15
Serum creatinine (μmol/l)	109 [89; 140]	100 [86; 143]	0.84
Anti-PLA2R1 titer (RU/ml)	3 [1–15]	2 [0–18]	0.86
CD19 (cell/μl)	0 [0–19]	0 [0–0]	0.46
Serum rituximab level (μg/ml)	3.59 [0.14–7.69]	2.27 [0.59–8.32]	0.74
**CHARACTERISTICS AT MONTH 6**			
Proteinuria (g/g)	5.6 [2.0–6.4]	1.5 [0.6–4.3]	0.13
Serum creatinine (μmol/l)	104 [86–159]	102 [84–136]	0.29
Anti-PLA2R1 titer (RU/ml)	2 [0–68]	0 [0–10]	0.43
CD19 (cell/μl)	75 [57–89]	2 [0–41]	0.006^*^
**CHARACTERISTICS AT MONTH 12**			
Proteinuria (g/g)	1.7 [0.7–5.8]	0.6 [0.2–3.4]	0.03^*^
Serum creatinine (μmol/l)	94 [84–110]	106 [88–129]	0.39
Serum albumin (g/dl)	3.0 ± 0.8	3.1 ± 0.6	0.88
Anti-PLA2R1 titer (RU/ml)	10 [0–27]	1 [0–3]	0.27
**CHARACTERISTICS BEFORE TREATMENT MODIFICATION**			
Remission after one course	8/10 (80%)	27/34 (79%)	>0.99
Relapse	5/10 (50%)	3/34 (9%)	0.009^*^
Serum creatinine (μmol/l)	101 [86; 130]	107 [88; 165]	0.75
Anti-PLA2R1 titer (RU/ml)	8 [0; 34]	0.5 [0; 15]	0.09
Proteinuria (g/g)	3.5 [1.6; 7.1]	1.7 [0.2; 1.7]	0.0004^*^
Time of follow-up	32 [13.5; 92.0]	30.0 [24.0; 96.0]	0.90
2nd course of rituximab required	7/10 (70%)	10/34 (29%)	0.03^*^

Only one patient in each group presented a drug infusion reaction that did not require treatment discontinuation.

### Relapses and Anti-Rituximab Antibodies

Eight patients relapsed after achieving remission (*n* = 35), with a median time to relapse of 15 months [10.5; 24]. Five of these patients had anti-rituximab antibodies (Figure 1). In contrast, among the 27 patients who did not relapse, only three patients had anti-rituximab antibodies and two had persistent anti-rituximab antibodies (*p* = 0.007) (Table 4).

**Table 4:** Baseline characteristics and outcome data in relapsing and non-relapsing patients.

**Patients characteristics**	**Relapse** ***n* = 8**	**No relapse** ***n* = 27**	***p-*value**
**BASELINE CHARACTERISTICS**			
Age	63 ± 13	62 ± 14	0.85
Sex Ratio (F/M)	3/5	9/18	0.99
Proteinuria (g/g)	6.3 [5.2–9.4]	5.4 [4.5–7.6]	0.29
Serum albumin (g/dl)	2.1 ± 0.9	2.1 ± 0.7	0.77
Serum creatinine (μmol/l)	126 [94–146]	104 [76–98]	0.40
Anti-PLA2R1 titer (RU/ml)	102 [25–171]	152 [60–253]	0.33
**CHARACTERISTICS AT MONTH-3**			
Proteinuria (g/g)	5.9 [1.8; 6.9]	2.2 [0.9; 6.2]	0.02^*^
Serum creatinine (μmol/l)	116 [92; 240]	99 [79; 141]	0.16
Anti-PLA2R1 titer (RU/ml)	15 [0–18]	1 [0–14]	0.35
CD19 count (cell/μl)	0 [0–9]	0 [0–0]	0.03^*^
Serum rituximab level (μg/ml)	2.70 [0.01–7.41]	2.24 [0.28–9.39]	0.62
**CHARACTERISTICS AT MONTH-6**			
Proteinuria (g/g)	3.3 [1.6; 7.3]	1.4 [0.5; 1.9]	0.04^*^
Serum albumin (g/dl)	3.0 ± 1.0	3.2 ± 0.6	0.43
Serum creatinine (μmol/l)	105 [104; 157]	93 [82; 133]	0.08
Anti-PLA2R1 titer (RU/ml)	5 [0–30]	0 [0–2]	0.04^*^
CD19 count (cell/μl)	5 [2–115]	3 [0–63]	0.60
Anti-rituximab antibodies	5/8 (63%)	3/27 (11%)	0.007^*^

There was no significant difference between patients with or without relapse for age, proteinuria and PLA2R1-antibodies titers at baseline (Table 4). Relapsing patients were more likely to have higher CD19 count at month-3 (0 [0; 9] vs. 0 [0; 0] *p* = 0.03), higher proteinuria at month-3 (5.9 [1.8; 6.9] vs. 2.2 [0.9; 6.2] *p* = 0.02) and month-6 (3.3 [1.6; 7.3] vs. 1.4 [0.5; 1.9] *p* = 0.04), higher anti-PLA2R1 antibodies titer at month-6 (5 [0; 30] vs. 0 [0; 2] *p* = 0.04) and anti-rituximab antibodies at month-6 [5/8 (63%) vs. 3/27 (11%) *p* = 0.007] (Table 4).

Because the analysis of the relapse data is too complicated for logistic regression and each relapse occurred at different time points, we performed a time-to-event analysis of renal survival. Renal event was defined by achieving relapse within 2 years after the first course of rituximab. The rate of relapse was significantly higher for patients with anti-rituximab antibodies (*p* < 0.001) (Figure 2D).

### Neutralizing Effect of Anti-Rituximab Antibodies

The minimal cytotoxic concentration, defined, as the lowest dose required producing ≥50% of B-cells cytotoxicity, was 50 ng/ml for all anti-CD20 monoclonal antibodies (Supplementary Figure 5).

**Figure 3:**
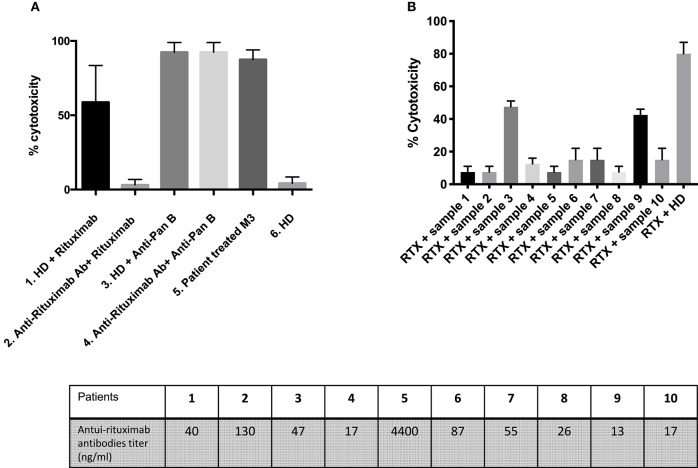
Detection of neutralizing anti-rituximab antibodies. **(A)** Anti-rituximab antibodies neutralized complement dependent cytotoxicity induced by rituximab. Purified B cells were incubated for 30 min with the following samples: (1) Serum from a healthy donor (HD) pre-incubated 2 h at room temperature at a ratio 1:2 with rituximab 50 ng/mL; (2) Serum from a patient with anti-rituximab antibodies (Patient 1) pre-incubated 2 h at room temperature at a ratio 1:2 with rituximab 50 ng/mL; (3) Serum from a healthy donor pre-incubated 2 h at room temperature at a ratio 1:2 with anti-pan B antibody 10 ng/ml (without specific for CD20) (Ingen^©^); (4) Serum from a patient with anti-rituximab antibodies pre-incubated 2 h at room temperature at a ratio 1:2 with anti-pan B antibody 10 ng/ml (Ingen^©^); (5) Serum from a patient treated with rituximab 3 months earlier (with residual rituximab concentration of 7.41 μg/ml) and no anti-rituximab antibody; (6) Serum from a healthy donor. The histogram shows the percent of lysed B-cells after adding rabbit complement. About 60–80% of B cells were lysed upon rituximab addition. Note that the percent of lysis decreased to 10% when rituximab was pre-incubated with anti-rituximab antibodies sera and that the inhibitory effect of anti-rituximab antibodies is specific for rituximab mediated lysis (no effect on pan-B mediated lysis). Error bars represent SD of the mean. The lysis is positive when more that 40% of the cells are lysed. **(B)** Screening of patients with anti-rituximab antibodies for inhibition of rituximab induced complement dependent cytotoxicity. The first 10 histograms represent each of 10 patients with anti-rituximab antibodies by ELISA at month-6. Note that only two patients (#3 and #9) presented non-neutralizing anti-rituximab antibodies. The last histogram represents serum from healthy donor pre-incubated 2 h at room temperature at ratio 1:2 with rituximab 50 ng/ml (positive control of lysis). *Mean statistically significant.

In a complement-dependent cytotoxicity assay, rituximab (50 ng/ml) spiked into healthy donor serum in the presence of rabbit complement induced 60% B-cell death while healthy donor serum alone had no effect (Figure 3A). The rituximab effect occurred at a concentration matching *in vivo* therapeutic conditions (7.4 μg/mL). When rituximab was spiked in patients' sera containing anti-rituximab antibodies, its effect was blocked for eight out of 10 patients (Figure 3B). In contrast, anti-rituximab antibodies did not prevent cell death induced by anti-pan B antibodies. We could notice that anti-rituximab antibodies titer did not correlate with neutralizing effect or transitory antibodies.

**Figure 4:**
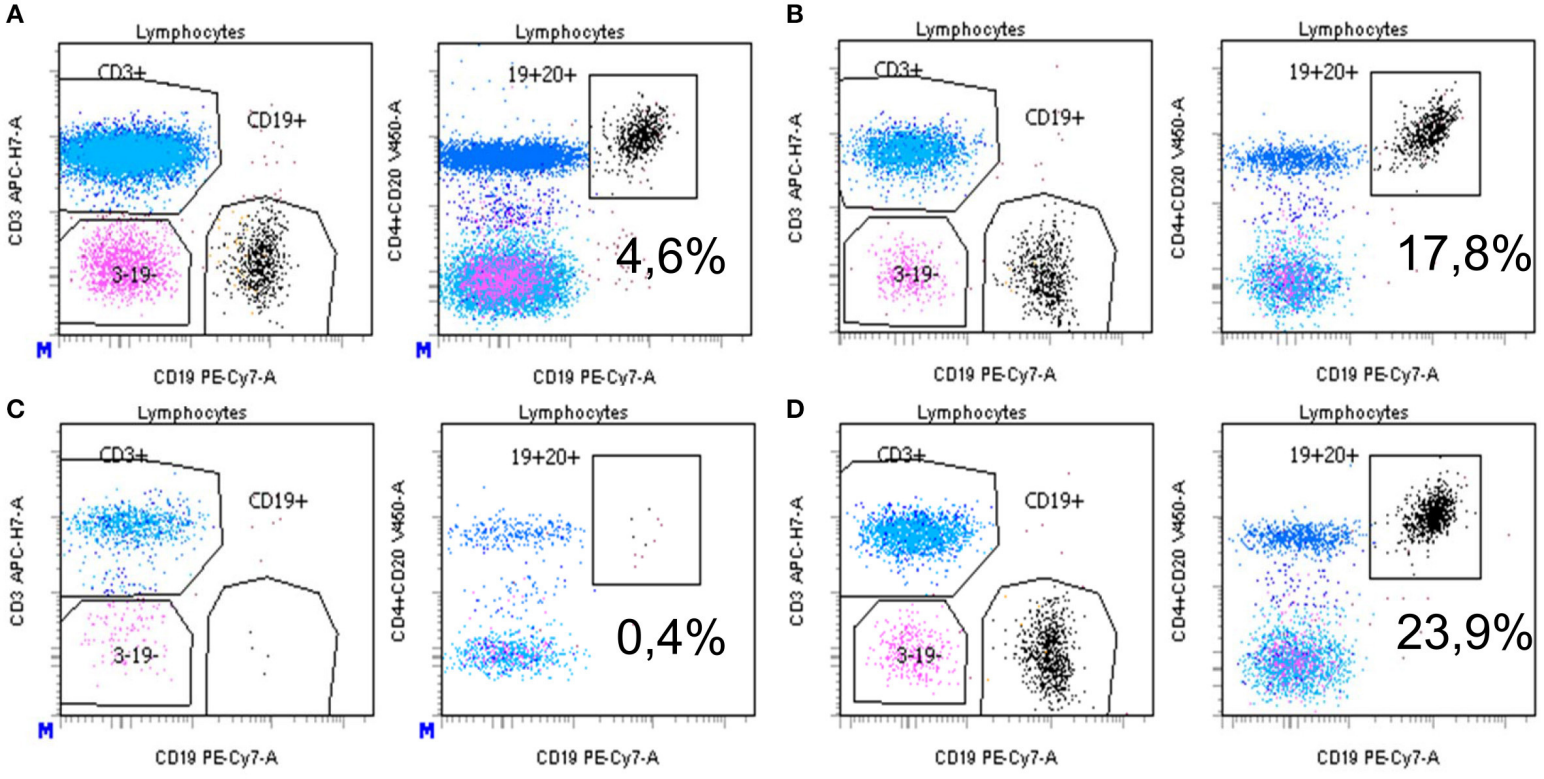
**(A–D)** Detection of neutralizing anti-rituximab antibodies: inhibition of antibody-dependent complement independent cell cytotoxicity induced by rituximab. After isolation of PBMC by Ficoll Hypaque centrifugation, cells were pre-incubated with complement-deprived normal human serum **(A,C)** or human serum containing anti-rituximab antibodies **(B,D)**, then incubated overnight with rituximab at 2 different concentrations (**A,B** 30 ng/ml; **C,D** 50 ng/mL). Then, cells were washed, labeled with monoclonal antibodies and subjected to flow cytometry analysis as described in the Methods section. Results are expressed as the % of CD19+CD20+ B cells among total lymphocytes. Gating is vs. CD3+ cells (left) or vs. CD4+ CD20+ cells (right). Equal number of PBMC was acquired in each condition. *Mean statistically significant.

In a complement-independent B-cell cytotoxicity assay, we tested the effect of rituximab (30 and 50 ng/ml) on the induction of B-cell apoptosis in the absence of complement activation, by flow cytometry. Overnight incubation of rituximab spiked into complement-depleted sera from healthy donors added to a total lymphocyte population led to a decrease of CD19+ cell counts as compared to incubation in the absence of rituximab (Figures 4A,C). In contrast, when rituximab was spiked in a representative complement-depleted patient's serum containing anti-rituximab antibodies, the proportion of CD19+ cells increased from 4.6 to 17.8% (Figure 4B, 30 ng/ml of rituximab) and from 0.4 to 23.9% (Figure 4D, 50 ng/ml of rituximab). Collectively, these studies showed that eight of the 10 patients with anti-rituximab antibodies produced neutralizing antibodies at concentrations sufficient to block the cytotoxic effects of rituximab with and without complement activity.

### Cross-Reactivity of Neutralizing Anti-Rituximab Antibodies With New Anti-CD20 Monoclonal Antibodies

**Figure 5:**
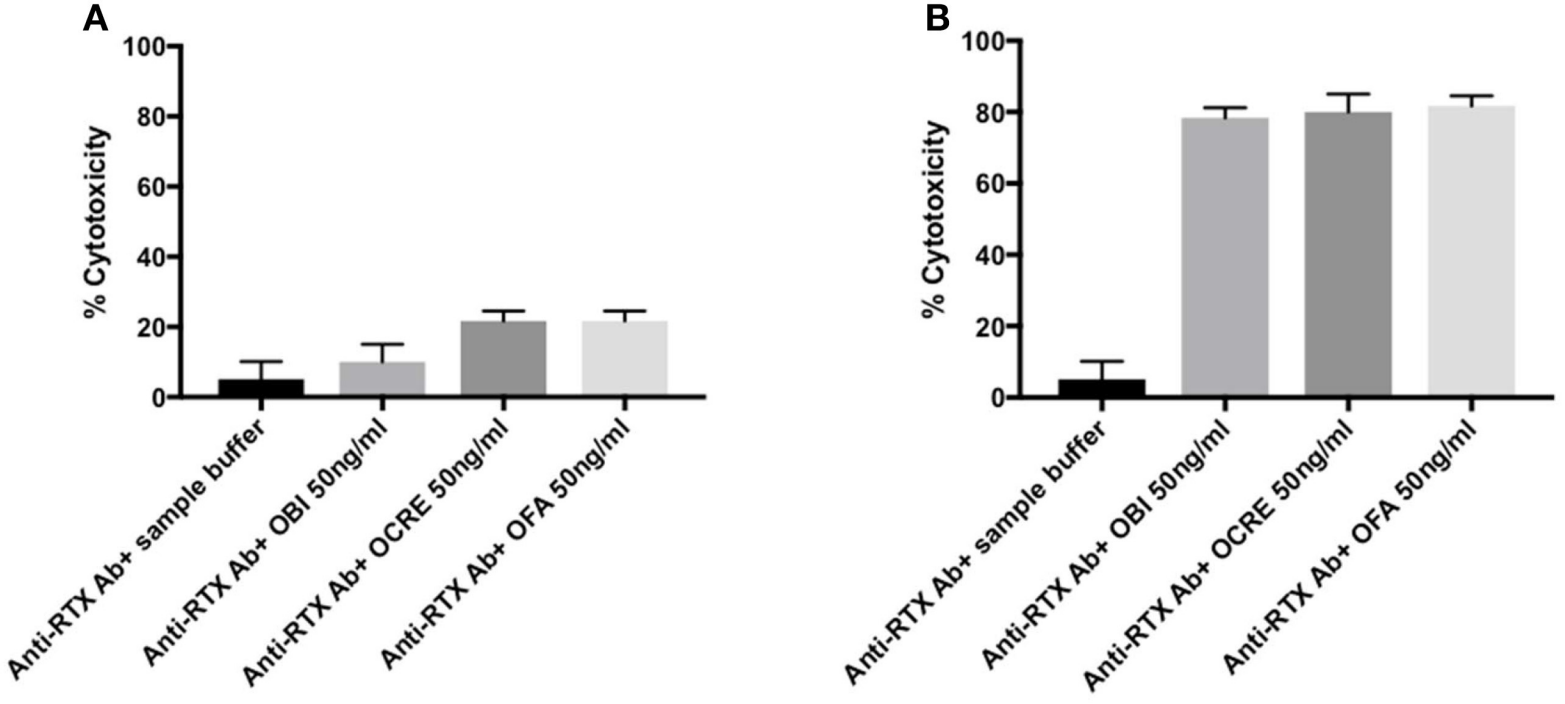
**(A,B)** Cross reactivity for humanized and fully human anti-CD20 monoclonal antibodies in rituximab-immunized patients. **(A)** For patients 1 and 2, anti-rituximab antibodies neutralized both obinutuzumab, ocrelizumab, and ofatumumab. Purified B cells were incubated for 30 min with the following samples: Serum from patients 1 and 2 pre-incubated 2 h at room temperature at a ratio 1:2 with sample buffer or anti-CD20 monoclonal antibody (obinutuzumab, ocrelizumab, or ofatumumab) at 50 ng/ml. Histogram show the mean value for patients 1 and 2 and standard deviation. **(B)** For patients 3–10, there was no cross-reactivity between new anti-CD20 therapies and anti-rituximab antibodies. Purified B cells were incubated for 30 min with the following samples: Serum from patients 3–10 pre-incubated 2 h at room temperature at a ratio 1:2 with sample buffer or anti-CD20 monoclonal antibody (obinutuzumab, ocrelizumab, or ofatumumab) at 50 ng/ml. Histogram show the mean value for patients 3 to 10 and standard deviation. RTX, rituximab; Anti-RTX Ab, anti-rituximab antibodies; OBI, obinutuzumab; OCRE, ocrelizumab; OFA, ofatumumab.

We tested whether anti-rituximab antibodies inhibited cytotoxicity of humanized and fully human anti-CD20 monoclonal antibodies. Results are summarized in Figures 1, 5A,B.

Two patients' profiles were observed. Anti-rituximab antibodies from patients 1 and 2 blocked B-cell cytotoxicity for obinutuzumab, ocrelizumab, and ofatumumab (Figure 5A) whereas anti-CD20 monoclonal antibodies efficacy was not impaired for the other eight patients (Figure 5B).

### Evolution and Personalized Care According to Rituximab Immunization

Patients' evolution is summarized in Figure 1.

We adapted our therapeutic strategy to this profile: using ofatumumab or another immunosuppressive therapy when anti-rituximab antibodies were detected.

**Figure 6:**
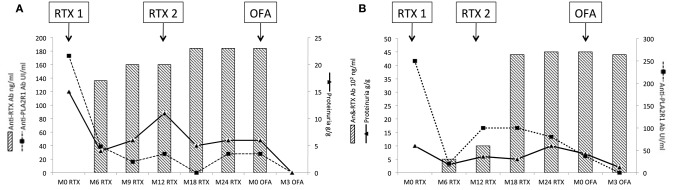
Evolution of rituximab-resistant patients treated with ofatumumab. Patients 4 and 5 were resistant after two courses of rituximab and developed neutralizing anti-rituximab antibodies. Anti-rituximab antibodies did not neutralize *in vitro* B-cells cytotoxicity for ofatumumab. These two patients were treated with ofatumumab (300 mg on day 1 and 1,000 mg on day 8 and day 21). **(A)** Evolution for patient 4: patient 4 developed anti-rituximab antibodies (17 ng/mL at month-6 and reached 23 ng/ml at month-24). After ofatumumab infusions, anti-rituximab antibodies get negative within 3 months. Anti-PLA2R1 antibodies became also negative and proteinuria decreased to 0.4 g/g. **(B)** Evolution for patient 5: patient 5 developed anti-rituximab antibodies (500 ng/ml at month-6 and reached 4,500 ng/mL at month-24). After ofatumumab infusions, anti-rituximab antibodies were still detected and stable at 4,400 ng/ml at month-3. Anti-PLA2R1 antibodies became negative and proteinuria decreased to 2 g/g. RTX, rituximab; OFA, ofatumumab.

Patients 4, 5, 7, and 10 had anti-rituximab antibodies that did not cross-react with new anti-CD20 monoclonal antibodies and were treated with ofatumumab (300 mg on day 1 and 1,000 mg on day 8 ± day 21 according to clinical and immunological response) and cyclophosphamide when ofatumumab was not available. Patients 4 and 5 were resistant after two courses of rituximab; patient 7 relapsed after one course of rituximab. Those three patients achieved remission at month-3 after ofatumumab therapy. After ofatumumab, anti-rituximab antibodies disappeared at month-3 for patient 4 (Figure 6A) and were persistent at the same high titer for patient 5 (Figure 6B).

Patient 1 was treated with four courses of rituximab for MN relapses. During the last relapse, he developed primary biliary cirrhosis associated with anti-M2 and anti-gp120 antibodies. These antibodies were detected after his first relapse but before the onset of biliary cirrhosis symptoms and were negative at diagnosis. Due to cross-reactivity with new anti-CD20 monoclonal antibodies and the development of primary biliary cirrhosis, he was switched to azathioprine and finally achieved remission for his MN.

Patients 3 and 9 developed non-neutralizing anti-rituximab antibodies and achieved remission on rituximab therapy.

## Discussion

Anti-rituximab antibodies were detected in 23% of patients treated with rituximab for idiopathic MN and were associated with the need for at least two courses of rituximab for resistant MN or relapses. These results are similar to findings of antidrug antibodies following anti-TNFα therapy (31–35) and rituximab in ANCA-vasculitis, SLE (11, 36) and multiple sclerosis (37). As described by Fervenza et al., we found that anti-rituximab antibodies were not associated with lower remission (15) but we described their association with MN relapses. Conversely, we confirmed that residual serum rituximab levels at month 3 were associated with remission (20, 23). Initial CD19+ B cell depletion was seen in all patients; but in patients who developed anti-rituximab antibodies, or in patients with undetectable residual rituximab serum level at month-3 as we previously described (23), B cells started to recover earlier.

Anti-rituximab antibodies probably appear before month-6 but we are able to detect them only at month-6 on their free fraction. B cell depletion is possible until circulating rituximab persists. Between month-3 and month-6, anti-rituximab antibodies could block B cell depletion. In fact, in non-immunized patients, B cell depletion is prolonged until month-6 while in immunized patients, they recover B cells earlier. This might be associated with relapse or incomplete response to treatment while B cells repopulation might be a more intricate mechanism. In clinical practice in case of resistant or relapse MN, anti-rituximab should be considered to help optimized therapeutic strategy, some of them would not respond to a second course of rituximab.

We established with two different methods that anti-rituximab antibodies neutralized rituximab mediated B-cell depletion and could negatively affect clinical response. Unfortunately, we could not use samples from non-immunized patients as controls. Inhibition of rituximab activity could favor the persistence of pathogenic memory B-cells and induce disease relapse, with an earlier reconstitution of B-cell compartment (38). Distinctions have been made between non-neutralizing antibodies that do not inhibit the clinical effect of a drug and neutralizing antidrug antibodies. However, the presence of neutralizing antidrug antibodies is not always associated with a decreased therapeutic effect. Pharmacological efficacy depends on the balance between drug concentrations and antidrug-antibodies levels in some cases drug levels are sufficient to induce the therapeutic drug effect. By contrast, non-neutralizing antidrug antibodies, link to a portion far from the paratope of the drug molecule and does not neutralize its therapeutic activity (e.g., to the allotope). In such cases, the formation of antibodies is triggered by polymorphisms expressed in the constant portion of the immunoglobulin, which vary between individuals. The biologic effect of non-neutralizing antidrug antibodies is less well understood (39–42), but the formation of immune complexes may accelerate drug clearance by the reticuloendothelial system (42). However, our patients with non-neutralizing anti-rituximab antibodies showed a favorable outcome after two courses of rituximab.

It is reported that patients with antidrug antibodies present more infusion-related reactions (29), in this study we did not confirm this finding as reported in two other studies (11, 40).

If we consider that immunogenicity is an important factor that should be considered in the overall treatment strategy, we should take actions to reduce antidrug antibodies formation: modifying drug administration; increasing dose; decreasing immunogenicity by adding immunosuppressive agents to the regimen or using new drugs which are supposed to be less immunogenic such as humanized or fully human monoclonal antibodies. In this perspective, we evaluated *in vitro* whether anti-rituximab antibodies developed in some of MN patients could inhibit B-cell depletion by three new humanized anti-CD20 therapies. To our knowledge, this is the first study looking at cross-reactivity between antidrug antibodies developed in patients treated with rituximab and new anti-CD20 molecules. For two patients, anti-rituximab antibodies blocked both obinutuzumab, ocrelizumab, and ofatumumab B-cells cytotoxicity, suggesting an anti-idiotype activity (43). In contrast, non-neutralizing antidrug antibodies might bind to allotopes and human neo-antigens at the hinge of fusion proteins. Three of the MN patients with neutralizing anti-rituximab antibodies that did not interfere with new anti-CD20 monoclonal antibodies activity were successfully treated with ofatumumab. One patient was treated with cyclophosphamide because ofatumumab was not available. But using new anti-CD20 therapy should be considered to avoid side effects (18). After ofatumumab infusion, anti-rituximab antibodies disappeared for two patients, and were persistent at the same titer for the other one. This suggests plasma cells secreting anti-rituximab antibodies are long memory plasma cells or derived from B cells which may have lost CD20 expression as reported in lymphoma where CD20 expression level has been related to acquired rituximab resistance (25). Case reports and one study reported ofatumumab-effectiveness in children with resistant nephrotic syndrome (44, 45). To our knowledge, study or report on adult nephrotic syndrome and MN treated with ofatumumab are lacking. In these articles, ofatumumab use is not supported by an immunological rationale or based on drug monitoring.

The main limitation of our study is that it is a monocentric retrospective study analyzing a relatively small number of patients. Nevertheless, our study remains original and innovative. Drug monitoring and development of antidrug antibodies have been well-described in anti-TNFα but studies on rituximab are recent and rare in nephrology field (16, 23). Our work is the first to suggest the value of immune-monitoring in adapting the therapeutic strategy in MN, particularly in resistant or relapsing cases. First, neutralizing anti-rituximab antibodies are not rare and their presence at month-6 is associated with subsequent relapses. Anti-rituximab antibodies might be an useful biomarker adding to residual rituximab monitoring (23), anti-PLA2R1 antibodies titer in anti-PLA2R1 antibodies related MN (46), epitope spreading (47, 48) to predict clinical outcomes, and it need to be tested in prospective studies. Secondly, rituximab immuno-monitoring might also be a helping tool for PLA2R1-negative MN patients. Then, anti-rituximab antibodies may or may not interfere with new humanized anti-CD20 monoclonal antibodies, allowing tailored rescue therapies. All last, basing on immunological and clinical arguments, new humanized anti-CD20 seems to be a satisfying therapeutic alternative in adult patients with rituximab-resistant or relapsing MN or rituximab intolerance like serum sickness. Further studies are needed to develop personalized therapeutic strategies in primary MN based on drug monitoring and immunogenicity testing.

## Data Availability Statement

The datasets generated for this study are available on request to the corresponding author.

## Ethics Statement

The studies involving human participants were reviewed and approved by CPP CHU Nice. Written informed consent to participate in this study was provided by all participants.

## Author Contributions

SB-S, BS-P, and VE conceived and designed the study and wrote the paper. SB-S and BS-P analyzed and interpreted the data. All authors contributed reagents, materials, analysis tools, or data.

### Conflict of Interest

The authors declare that the research was conducted in the absence of any commercial or financial relationships that could be construed as a potential conflict of interest.

## Supplementary Material

Supplementary Figure 1 Correlation of serum rituximab level at month 3 and proteinuria at month 6.

Supplementary Figure 2 Evolution of proteinuria **(A)** and anti-PLA2R1 titer **(B)** according to therapeutic strategy required: one course of rituximab needed *n* = 27 and new therapeutic strategy required *n* = 17.

Supplementary Figure 3 Evolution of proteinuria **(A)**, anti-PLA2R1 titer **(B)**, and CD19 count **(C)** in resistant (*n* = 9) and non-resistant (*n* = 35). Membranous Nephropathy.

Supplementary Figure 4 Evolution of proteinuria **(A)**, anti-PLA2R1 titer **(B)**, and CD19 count **(C)** in relapsing (*n* = 8) and non-relapsing (*n* = 27). Membranous Nephropathy (excluding resistant MN).

Supplementary Figure 5 Determination of anti-CD20 monoclonal antibody minimal cytotoxic concentration for *in vitro* complement-dependent cytotoxicity assay. For both, the minimal concentration required to produce ≥50% of cytotoxicity was 50 ng/ml. **(A)** Assessment for rituximab. **(B)** Assessment for obinutuzumab. **(C)** Assessment for ocrelizumab. **(D)** Assessment for ofatumumab. RTX, rituximab; OBI, obinutuzumab; OCRE, ocrelizumab; OFA, ofatumumab; Ab, antibodies.
