# Supplementary figures and images for: Neutralizing Anti-Rituximab Antibodies and Relapse in Membranous Nephropathy Treated With Rituximab
Source: Front Immunol. 2020 Jan 13;10:3069. doi: 10.3389/fimmu.2019.03069 (PMC6970431; doi:10.3389/fimmu.2019.03069)

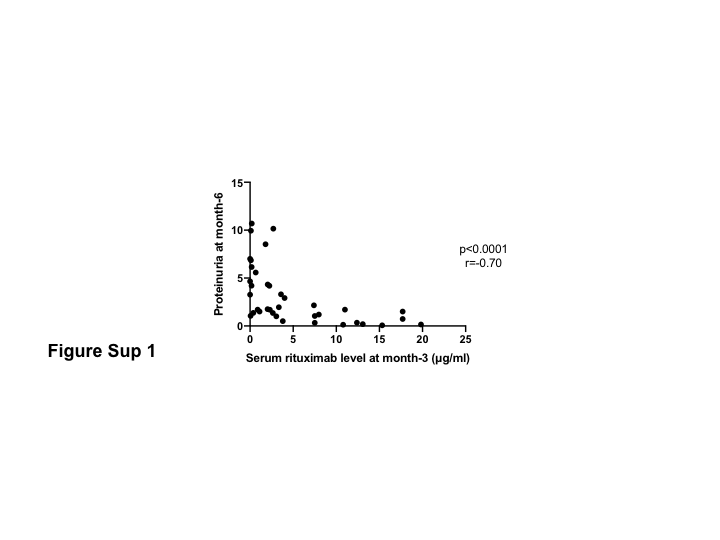

Supplement: Supplementary Figure 1 — Correlation of serum rituximab level at month 3 and proteinuria at month 6. [file Image_1.TIFF]

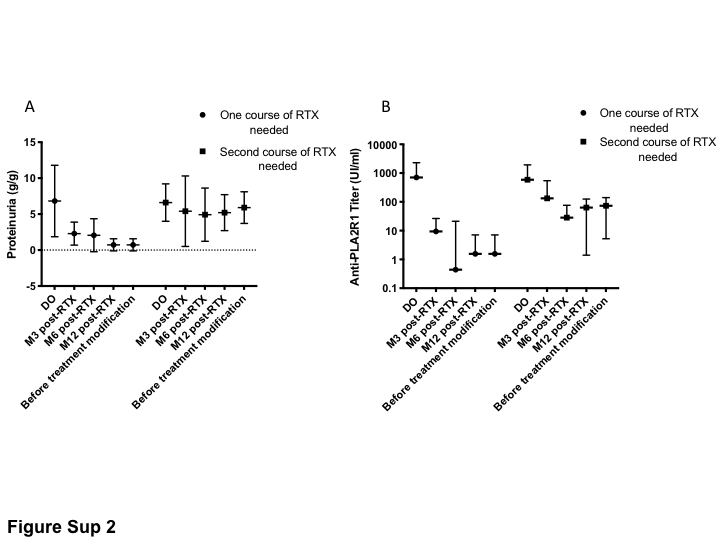

Supplement: Supplementary Figure 2 — Evolution of proteinuria (A) and anti-PLA2R1 titer (B) according to therapeutic strategy required: one course of rituximab needed n = 27 and new therapeutic strategy required n = 17. [file Image_2.TIFF]

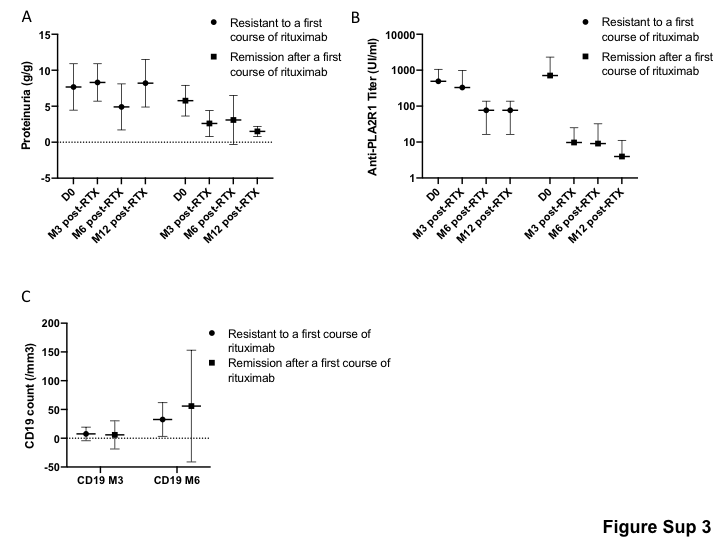

Supplement: Supplementary Figure 3 — Evolution of proteinuria (A), anti-PLA2R1 titer (B), and CD19 count (C) in resistant (n = 9) and non-resistant (n = 35). Membranous Nephropathy. [file Image_3.TIFF]

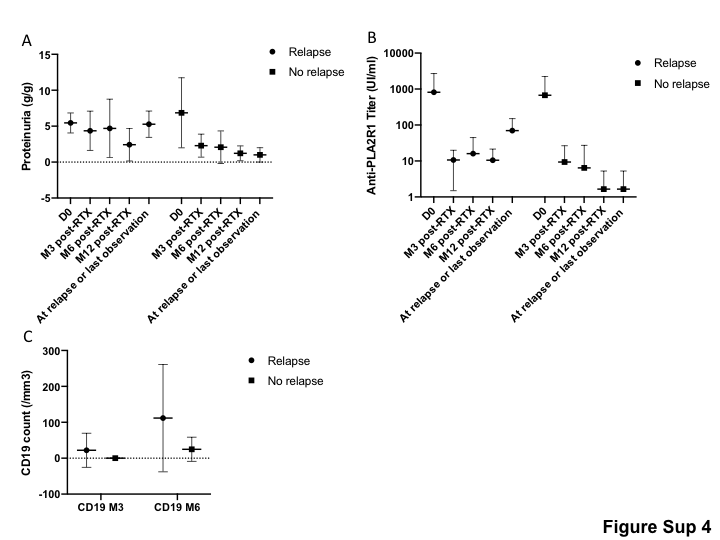

Supplement: Supplementary Figure 4 — Evolution of proteinuria (A), anti-PLA2R1 titer (B), and CD19 count (C) in relapsing (n = 8) and non-relapsing (n = 27). Membranous Nephropathy (excluding resistant MN). [file Image_4.TIFF]

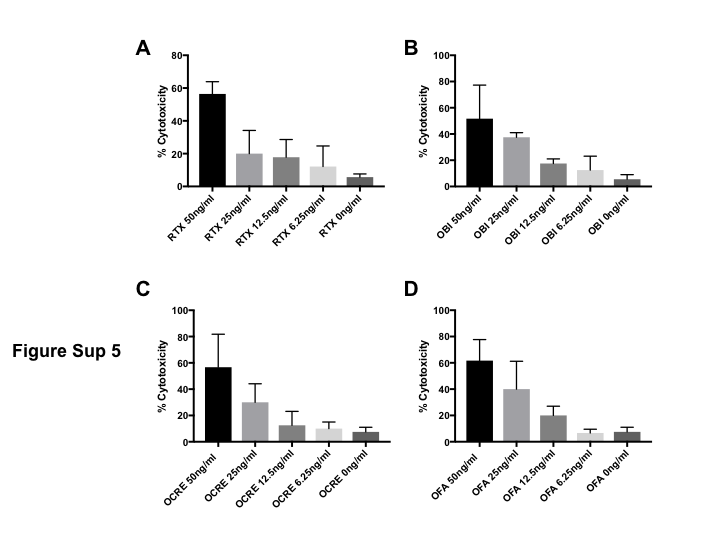

Supplement: Supplementary Figure 5 — Determination of anti-CD20 monoclonal antibody minimal cytotoxic concentration for in vitro complement-dependent cytotoxicity assay. For both, the minimal concentration required to produce ≥50% of cytotoxicity was 50 ng/ml. (A) Assessment for rituximab. (B) Assessment for obinutuzumab. (C) Assessment for ocrelizumab. (D) Assessment for ofatumumab. RTX, rituximab; OBI, obinutuzumab; OCRE, ocrelizumab; OFA, ofatumumab; Ab, antibodies. [file Image_5.TIFF]
